# VDAC1 Upregulation Induces Hyperexcitability of Nociceptive Sensory Neurons Via Enhanced Mitochondrial Atp Efflux in Neuropathic Pain

**DOI:** 10.1002/advs.202523223

**Published:** 2026-07-07

**Authors:** Fengrun Sun, Ning Yu, Liang Yu, Xiaofen Tian, Yujia Ma, Yikuan Xie, Tao Wang, Chao Ma

**Affiliations:** ^1^ State Key Laboratory of Common Mechanism Research for Major Diseases Department of Human Anatomy Histology and Embryology Neuroscience Center Joint Laboratory of Anesthesia and Pain Institute of Basic Medical Sciences Chinese Academy of Medical Sciences School of Basic Medicine Peking Union Medical College Beijing China; ^2^ Department of Anatomy Key Laboratory of Human Brain Bank for Functions and Diseases of Department of Education of Guizhou Province Guizhou Medical University Guiyang Guizhou China; ^3^ National Human Brain Bank for Development and Function Beijing China; ^4^ Chinese Institute for Brain Research Beijing China

**Keywords:** intracellular ATP, mitochondrion, neuronal excitability, neuropathic pain, VDAC1

## Abstract

Neuropathic pain results from aberrant sensory processing following nerve injury. Voltage‐dependent anion channels (VDACs), a family of ATP gatekeepers in the outer mitochondrial membrane, are central to cellular metabolism and action potential generation, yet their roles in peripheral neuropathic pain remain poorly defined. Here, we demonstrate that among the VDAC isoforms, VDAC1 is predominantly expressed in dorsal root ganglion (DRG) nociceptive neurons and is markedly upregulated in a mouse model of chronic constriction injury (CCI). Functionally, VDAC1 promotes neuropathic pain by increasing mitochondrial ATP permeability, thereby elevating cytoplasmic ATP levels and enhancing neuronal excitability. Genetic or pharmacological inhibition of VDAC1 reduced cytoplasmic ATP, suppresses neuronal hyperexcitability, and alleviates pain behaviors. Collectively, these findings indicate the role of VDAC1 in ATP dynamics and neuronal excitability, highlighting it as a potential therapeutic target for neuropathic pain.

## Graphical abstract

The mechanism diagram of VDAC1 mediating neuronal excitability and neuropathic pain. Briefly, VDAC1 is expressed in DRG neurons and is upregulated following CCI‐induced neuropathic pain. This upregulation enhances ATP transport from mitochondria to the cytoplasm in sensory neurons, leading to increased neuronal excitability and pain behavior. Genetic knockdown of *Vdac1* or pharmacological inhibition of VDAC1 alleviates neuropathic pain.

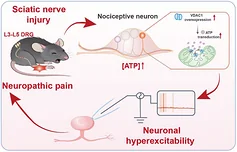

## 1 Introduction

Neuropathic pain originates from damage or dysfunction of the nervous system, resulting in an abnormal processing of sensory information [1]. The peripheral mechanisms underlying neuropathic pain are primarily caused by changes in the peripheral nervous system (PNS), particularly within primary afferent nociceptive neurons in the dorsal root ganglia (DRG). These neurons are predominantly small‐diameter primary sensory neurons and comprise multiple subpopulations, including peptidergic neurons marked by calcitonin gene‐related peptide (CGRP) and non‐peptidergic neurons labeled by isolectin B4 (IB4). In addition, transient receptor potential vanilloid 1 (TRPV1) is highly enriched in a subset of nociceptive DRG neurons [2, 3]. Following nerve injury, these nociceptors undergo various changes, such as altered ion channel expression, heightened neuronal excitability, and the development of aberrant synaptic plasticity. These alterations in turn contribute to the hallmark features of neuropathic pain, including hyperalgesia and allodynia [4, 5]. While the specific manifestations of neuropathic pain are diverse, the underlying increase in sensory neuronal excitability serves as a core mechanism. It is directly indicated by the abnormal firing of action potential (AP), which further contributes to the persistence and intensification of pain, and makes it a critical target for therapeutic interventions [6].

ATP plays a fundamental role in the generation and propagation of APs, such as supporting both the maintenance of ionic gradients and the function of membrane proteins that contribute to action potential dynamics, which are crucial for transmitting pain sensory information. The electrochemical gradients across the neuronal membrane, particularly for sodium (Na^+^), potassium (K^+^), and calcium (Ca^2^^+^) ions, are maintained by ATP‐dependent ion pumps, most notably the Na^+^/K^+^‐ATPase [7]. This creates the resting membrane potential necessary for action potential initiation and ensures the rapid repolarization of the membrane after each spike. A previous study has found that inhibition of DRG neuronal K_ATP_ channels leads to depolarization, aberrant firing, and increased neurotransmission [8]. Moreover, the recovery of the ion balance post‐activation again requires the activity of Na^+^/K^+^‐ATPase, emphasizing ATP's continuous role in resetting neuronal excitability.

The voltage‐dependent anion channel (VDAC) family comprises a group of highly conserved pore‐forming proteins located in the outer mitochondrial membrane (OMM), it is essential for regulating the flow of ions and metabolites between mitochondria and the cytosol [9, 10]. In mammals, the VDAC family consists of three main isoforms: VDAC1, VDAC2, and VDAC3. VDACs are crucial for mitochondrial function, energy metabolism, apoptosis, and calcium signaling, serving as key integrators of metabolic and survival pathways. Among the isoforms, VDAC1 is the most abundant and extensively studied member. The expression of VDAC1 is 10 times more abundant than VDAC2, and 100 times more prevalent than VDAC3 in HeLa cells [11]. It forms a large β‐barrel hydrophilic channel, controlling the permeability of ions and small hydrophilic molecules through allosteric regulation under different mitochondrial membrane potentials (MMPs). In physiological conditions, VDAC1 allows the passage of ionic metabolites (pyruvate, glutamate, succinate, malate), lipids, and inorganic ions (K^+^, Na^+^, Ca^2+^, chloridion (Cl^−^)) [12].

In the central nervous system (CNS), VDAC1 is expressed in energy‐demanding brain regions such as the hippocampus, cortex, cerebellum, and substantia nigra areas, and has critically emerged as a key player in the pathophysiology of various neurological disorders. Increasing evidence has linked VDAC1 dysregulation to neurodegenerative diseases such as Alzheimer's disease, Parkinson's disease, and amyotrophic lateral sclerosis [13, 14, 15]. Abnormal expression or oligomerization of VDAC1 has been associated with oxidative stress, mitochondrial dysfunction, and impaired energy metabolism, which are the hallmarks of neuronal degeneration. Moreover, VDAC1 interacts with key apoptotic and neuroinflammatory regulators, including members of the Bcl‐2 family and hexokinase, highlighting its involvement in both cell survival and death signaling pathways [16, 17]. Previous evidence has shown that VDAC1 is also expressed in the spinal cord. Microglial VDAC1 in the spinal cord mediates pain caused by sleep deprivation in rat models, via its oligomerization and cell membrane transfer [18].

However, there are relatively few studies focusing on the role of peripheral nervous system VDAC1 expression and its role in the pathogenesis of neuropathic pain. Previous studies have validated that VDAC1 mediates the permeability of purine nucleotides from mitochondrial to cytoplasm of VDAC1 under physiological conditions [19, 20, 21]. The regulation is essential for maintaining cellular energy homeostasis and ATP utilization in sensory neurons, indicating VDAC1 may participate in the occurrence and progression of neuropathic pain via mediating ATP balance between the mitochondrion and cytoplasm. In this study, we concentrate on the critical gate‐keeping role of DRG VDAC1 in neuropathic pain, especially the effects on neuronal excitability.

## 2 Results

### 2.1 VDAC1 is Selectively Expressed in Nociceptors

In mammals, the VDAC family comprises three main isoforms: VDAC1, VDAC2, and VDAC3. These isoforms share structural similarities and exhibit partially overlapping functions. To investigate their expression in sensory neurons, we first isolated mouse lumbar DRG neurons and performed single‐cell qPCR to assess the expression of each VDAC isoform in distinct sensory neuron subtypes (Figure 1A). We used molecular markers including *Trpv1* and *Scn10a* for nociceptors (TRPV1^+^, Nav1.8^+^), *Ntrk2* for low‐threshold mechanoreceptors (LTMRs; TrkB^+^), and *Ntrk3* for proprioceptors (TrkC^+^). Our analysis revealed that *Vdac2* and *Vdac3* genes were widely expressed across all three types of sensory neuron subtypes, whereas *Vdac1* expression was predominantly restricted to nociceptive neurons, suggesting a more specific role for VDAC1 in pain signaling (Figure 1B). Then we confirmed the localization of VDACs in sensory neurons by performing immunostaining of VDACs with the pan‐neuronal marker PGP9.5. The results further indicated that VDAC1 was confined to a subset of sensory neurons, while VDAC2 and VDAC3 were ubiquitously distributed throughout the DRG neuronal population (Figure 1C).

**FIGURE 1:**
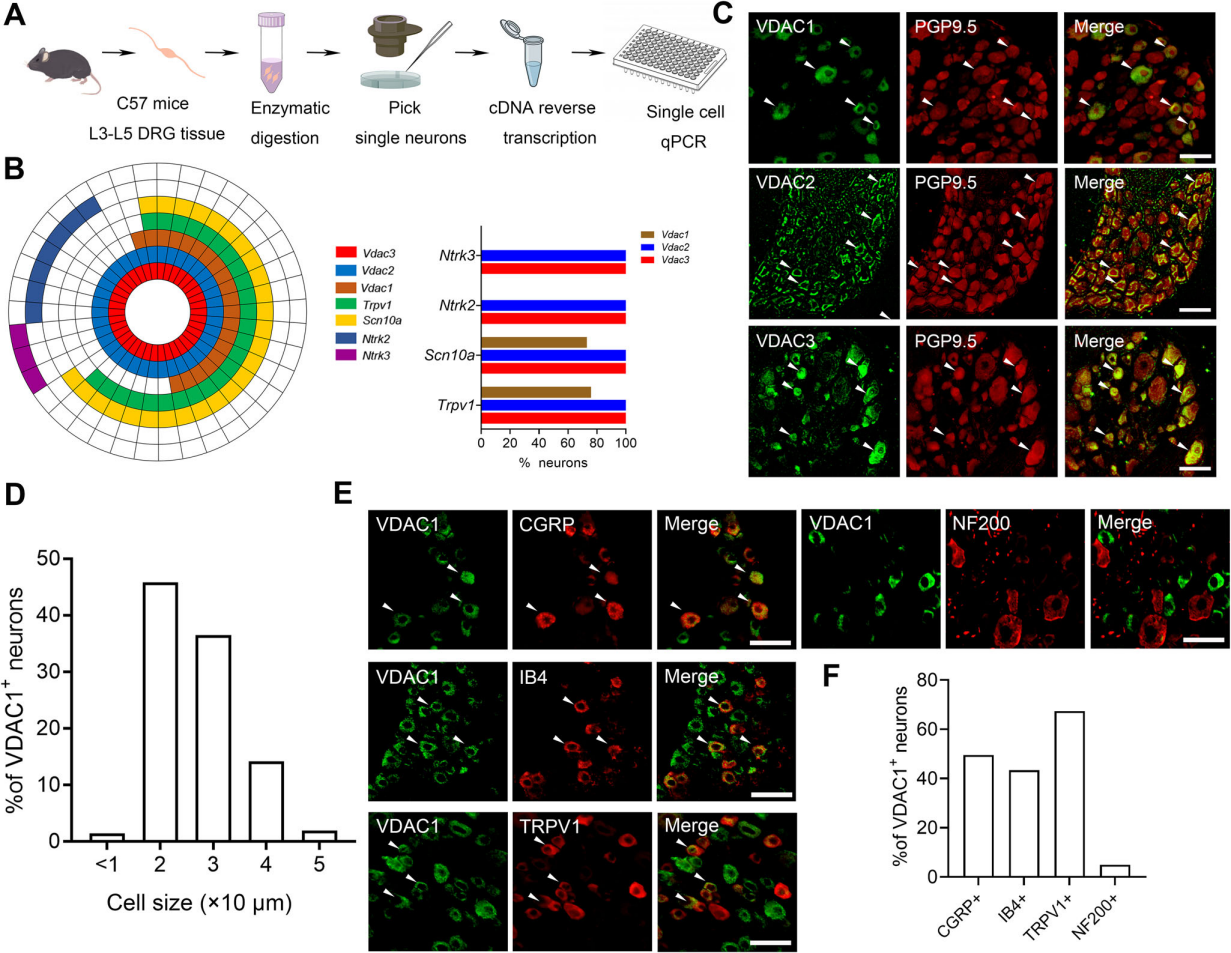
VDAC1 is selectively expressed in nociceptors. (A) The schematic diagram of the neuron isolation and single‐cell qPCR of *wt* mice L3‐L5 DRGs. (B) Single‐cell qPCR results of the expression of *Vdac1*, *Vdac2*, and *Vdac3* in *Trpv1^+^, Scn10a^+^, Ntrk2^+^, and Ntrk3^+^* DRG neurons. (C) Immunofluorescence staining of VDAC1, VDAC2, and VDAC3 in *wt* mouse DRG. Scale bars: 50 µm. (D) Cell size profiling of VDAC1^+^ neurons in *wt* mice DRG. (E‐F) Representative images of VDAC1 (green) with CGRP, IB4, TRPV1, and NF200 (red) in *wt* mice DRG, along with quantitative analysis of percentage overlap. Scale bars: 50 µm. n = 3 mice/group.

Because the broad ganglionic staining of VDAC2 and VDAC3 suggested that these isoforms might not be restricted to neurons, we next examined their expression in satellite glial cells. Double immunofluorescence staining with glutamine synthetase (GS), a marker of satellite glial cells, was performed in mouse DRG as well as in human sensory ganglia, including DRG and trigeminal ganglion (TG) tissues. VDAC1 showed no detectable co‐localization with GS in either mice or human sensory ganglia, indicating that it is not expressed in satellite glial cells. In contrast, both VDAC2 and VDAC3 exhibited clear colocalization with GS in mouse DRG and in human DRG and TG tissues, indicating that these two subtypes are also expressed in satellite glial cells (Figure S1A–C). These findings further demonstrate that VDAC1 might display the most restricted distribution in sensory ganglia, whereas VDAC2 and VDAC3 are more broadly distributed across both neuronal and glial populations.

Next, we examined VDAC1 expression in pain‐related sensory neurons. Consistent with single‐cell qPCR results, immunofluorescence staining demonstrated that VDAC1 was predominantly localized in small‐ and medium‐diameter nociceptive neurons (Figure 1D). Specifically, it showed strong co‐localization with nociceptive‐specific markers, including the peptidergic neuronal marker CGRP, the non‐peptidergic neuronal marker IB4, and the capsaicin (Cap)‐sensitive ion channel TRPV1. In contrast, VDAC1 was rarely co‐localized with the large‐diameter neuronal marker neurofilament 200 (NF200), with less than 5% overlap observed (Figure 1E,F). To extend these findings to humans, we performed immunofluorescence staining on human DRG and TG, both critical peripheral nervous system structures for sensory transmission. Similar to the mouse data, VDAC1 in human tissues exhibited robust co‐localization with CGRP, IB4, and TRPV1, further reinforcing its selective association with nociceptive sensory processing (Figure S2A–C). Given the broad neuronal distribution of VDAC2 and VDAC3 observed in mice, we further supplemented the human sensory ganglion analysis by performing co‐staining of these two isoforms with PGP9.5. Similar to the mouse results, both VDAC2 and VDAC3 were detected in PGP9.5^+^ neurons in human TG and DRG tissues (Figure S3A,B).

### 2.2 Sensory Neuronal VDAC1 Mediates Nociception

We next investigated the function of VDAC isoforms in pain modulation by delivering siRNAs targeting *Vdac1*, *Vdac2* and *Vdac3* into L3‐L5 DRGs of mice (Figure 2A). Cap was used to be intradermal (ID) injected into the hindpaw to evoke acute pain in mice. To control the mechanical effect of needle insertion and injection itself, an equal volume of vehicle was injected intradermally into the contralateral or matched hindpaw as a control. Behavior assessments revealed that compared to scramble RNA (scRNA) controls, si*Vdac1* administration significantly alleviated acute pain induced by Cap injection. In contrast, *siVdac2* or *siVdac3* had no observable effects on either mechanical allodynia or heat hyperalgesia (Figure 2B–E).

**FIGURE 2:**
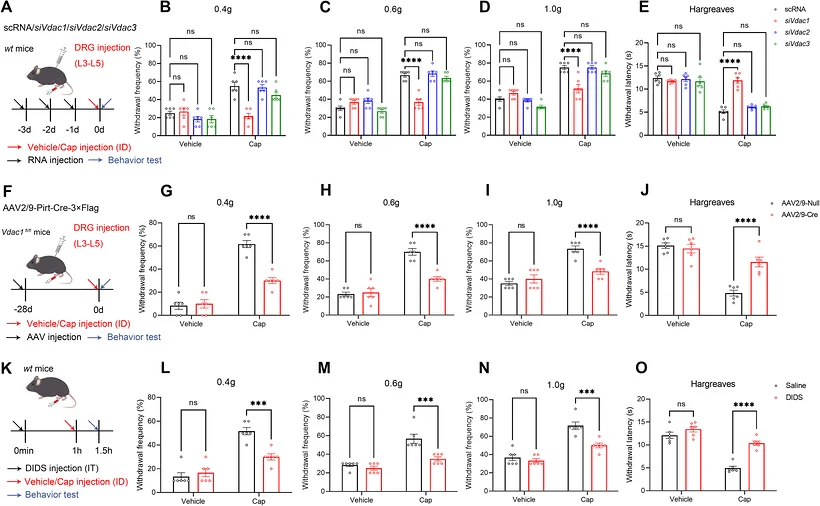
Sensory neuronal VDAC1 mediates acute pain. (A) The schematic diagram of scRNA or si*Vdac1/Vdac2/Vdac3* DRG injection and the workflow of drug administration and behavioral test. (B–E) Paw withdrawal frequency to 0.4 g, 0.6 g, and 1.0 g von Frey filaments and withdrawal latency in the Hargreaves test of mice after ID injection of Vehicle or Cap. n.s. not significant, ^****^p < 0.0001. Two‐way ANOVA test followed by Sidak's post hoc test. n = 6 mice/group. (F) The schematic diagram of AAV‐Cre or AAV‐Null DRG injection and the workflow of drug administration and behavioral test. (G–J) Paw withdrawal frequency to 0.4, 0.6, and 1.0 g von Frey filaments and withdrawal latency in the Hargreaves test of mice that received virus injected after ID injection of Vehicle or Cap. n.s. not significant, ^****^p < 0.0001. Two‐way ANOVA test followed by Sidak's post hoc test. n = 6 mice/group. (K) The schematic diagram of DIDS IT injection, along with the workflow of Cap administration and behavioral test. (L–O) Paw withdrawal frequency to 0.4, 0.6, and 1.0 g von Frey filaments and withdrawal latency in the Hargreaves test of mice that received DIDS or Saline administration (IT) after ID injection of Vehicle or Cap. n.s. not significant, ^***^p < 0.001, ^****^p < 0.0001. Two‐way ANOVA test followed by Sidak's post hoc test. n = 6 mice/group.

*Pirt* gene was originally described as being expressed in primary sensory DRG neurons, and the pirt‐Cre promoter has been widely used in pain studies to manipulate sensory neurons [22, 23, 24]. To further validate the role of sensory neuron VDAC1, we generated *Vdac1^fl/fl^* mice and injected AAV2/9‐pirt‐Cre‐3×Flag‐Null [adeno‐associated virus 2/9 (AAV2/9)‐Cre] or AAV2/9‐pirt‐Null (AAV2/9‐Null) into ipsilateral L3‐L5 DRGs 4 weeks prior to Cap injection (Figure 2F) [22]. AAV2/9‐Cre injection effectively reduced VDAC1 protein expression, as confirmed by immunoblotting and FLAG detection in DRG tissues from *Vdac1^fl/fl^* mice (Figure S4A–C). Strikingly, selective deletion of *Vdac1* significantly attenuated Cap‐induced mechanical allodynia and heat hyperalgesia, while leaving baseline sensory thresholds unchanged, whereas AAV2/9‐Null injection had no effect (Figure 2G–J and Figure S4D–G). In addition, we evaluated the effect of pharmacological inhibition of VDAC1 using 4,4'‐diisothiocyanatostilbene‐2,2'‐disulfonic acid (DIDS), an anion exchanger and Cl^−^ channel blocker, administered intrathecally (IT) (Figure 2K) [16, 18, 25, 26, 27]. Consistent with the genetic disruption of *Vdac1*, DIDS‐treated mice exhibited significantly increased tolerance to mechanical and thermal stimuli following Cap injection (Figure 2L–O). Together, these findings highlight a critical role for VDAC1 in nociceptive modulation without altering baseline sensory function.

### 2.3 VDAC1 Plays an Essential Role in the Development and Maintenance of Neuropathic Pain

To investigate the involvement of VDAC1 in the DRG during neuropathic pain, we employed the chronic constriction injury (CCI) model, which mimics peripheral neuropathic pain through partial ligation of the sciatic nerve. We first assessed VDAC1 expression in the L3‐L5 DRGs of mice following CCI surgery. Immunofluorescence staining revealed a robust upregulation of VDAC1 in DRG neurons after injury (Figure 3A,B). To quantitatively validate these findings, we performed both qPCR and western blot analyses and observed a significant increase in *Vdac1* mRNA and VDAC1 protein levels in the DRG after CCI (Figure 3C–E).

**FIGURE 3:**
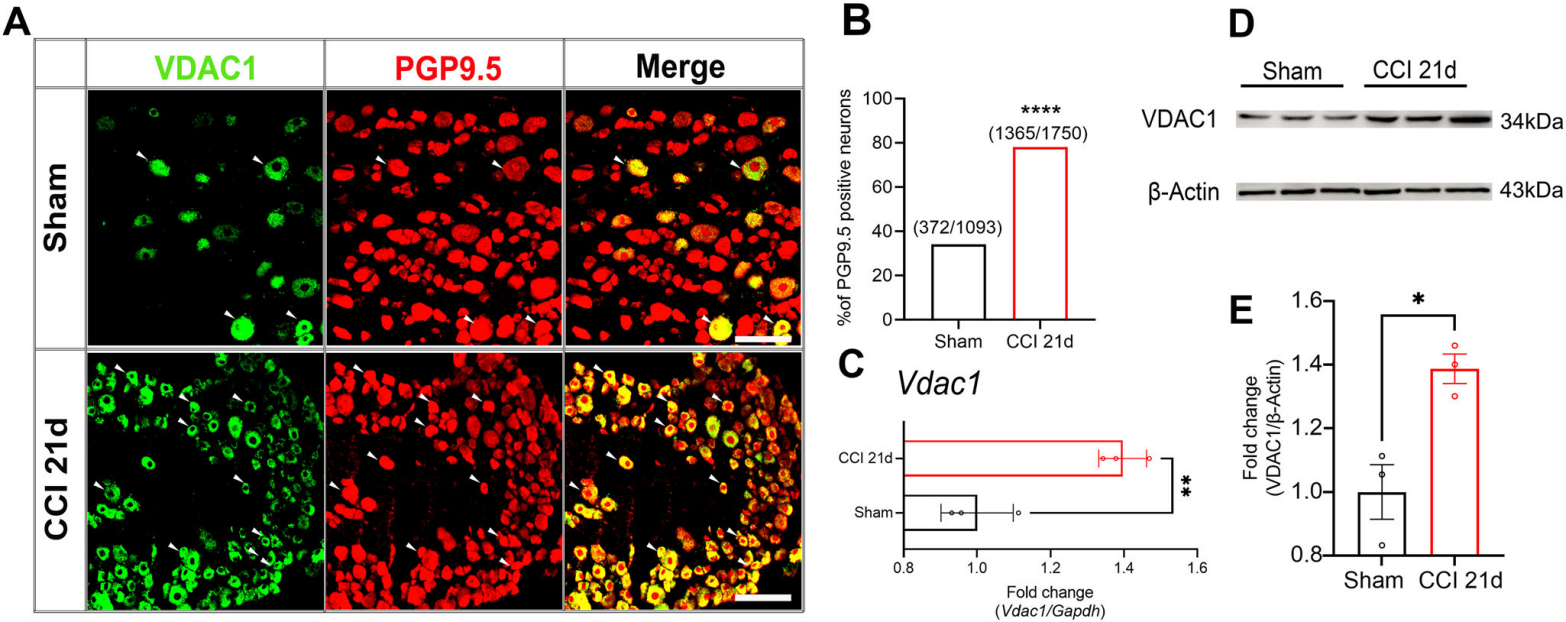
VDAC1 contributes to neuropathic pain induced by CCI. (A) Representative images of double staining of VDAC1 with PGP9.5 in DRG tissues from Sham and CCI 21 d mice. Scale bars: 50 µm. (B) Quantitative analysis of colocalization of VDAC1 and PGP9.5 in DRG tissues from Sham and CCI 21 d mice. ^****^p < 0.0001. Chi‐square test. n = 3 mice/group. (C) qPCR analyzes the mRNA level of *Vdac1* in L3‐L5 DRGs from Sham or CCI 21 d mice. ^**^p < 0.01. Student's *t*‐test. n = 3 mice/group. (D,E) Western blot analysis of VDAC1 in L3‐L5 DRGs from Sham or CCI 21 d mice. ^*^p < 0.05. Student's *t*‐test. n = 3 mice/group.

To further examine the cellular distribution of Vdac1 in injured sensory neurons, we reanalyzed a public CCI DRG single‐cell RNA sequencing (scRNA‐seq) dataset generated from *Pirt^EGFPf^* mice [28]. Neuronal subtype annotation was based on the original classification of the public CCI‐DRG scRNA‐seq dataset (GSE216039), including NP1 (*Mrgprd, P2rx3, Plxnc1*), NP3 (*Nppb, Sst, Il31ra*), PEP1 (*Tac1, Calca, Trpv1*), PEP2 (*Ntrk1, Nefh, Cacna1e*), injury‐associated NP neurons (*Gal, Sprr1a, Atf3*), and CCI‐induced clusters marked by injury‐response genes (*Atf3, Jun, Gal*, and *Sprr1a*) [28]. *Vdac1* was detectable across multiple nociceptor‐ and injury‐associated neuronal populations, supporting its expression in pain‐related sensory neurons (Figure S5A–D). However, sample‐level comparison between sham and CCI conditions did not reveal a consistent global increase in *Vdac1* mRNA in this dataset (Figure S5E). To further explore the potential mechanisms underlying VDAC1 upregulation after nerve injury, we performed predictive analyses from three aspects: transcription factors, signaling pathways, and post‐translational regulation. Our analysis further showed that Atf3, Jun, and Stat3 were upregulated after nerve injury in the public dataset. Previous studies have suggested that these transcription factors participate in pain‐related gene expression programs [29, 30, 31]. We therefore used the JASPAR database to examine whether they could potentially bind to the *Vdac1* promoter. The results suggest that there exist possible binding sites for Atf3, Jun, and Stat3 within the *Vdac1* promoter region, suggesting that these injury‐associated transcription factors may participate in upregulating VDAC1 expression after nerve injury‐induced pain (Figure S6A,B). At the pathway level, *Vdac1* expression showed a positive association with mitochondrial oxidative stress and ATP transport‐related transcriptional modules in our analysis. These findings are consistent with the basic function of VDAC1 as a major factor in cellular metabolism, indicating that CCI‐induced VDAC1 upregulation may occur in parallel with mitochondrial metabolic remodeling in injured nociceptive neurons (Figure S6C) [32].

To further examine the role of VDAC1 in neuropathic pain, we generated *Vdac1* knockout (KO) mice. Consistent with previous reports, the homozygotes of *Vdac1* KO mice were not viable beyond birth [33]. Therefore, heterozygous *Vdac1(±)* mice were used for subsequent experiments (Figure 4A–C). Since *Vdac1* is critical for mitochondrial functions, we first examined whether *Vdac1* knockdown could influence the mitochondrial status. We performed TEM, JC‐1 staining for MMP, and MitoROX staining on DRG neurons from naïve *Vdac1(+/+)* and *Vdac1(±)* mice. The intensity ratio of JC‐1 monomers to aggregates was used as an indicator of MMP, which served as a surrogate marker of mitochondrial metabolic activity. These results showed that there was no gross mitochondrial deficit in *Vdac1(±)* sensory neurons at baseline (Figure S7A–F). We have also explored the effects of *Vdac1* deficiency on the other VDAC subtypes. The results of qPCR and western blot showed there was no compensatory upregulation of *Vdac2* or *Vdac3* gene expression (Figure S7G–J).

**FIGURE 4:**
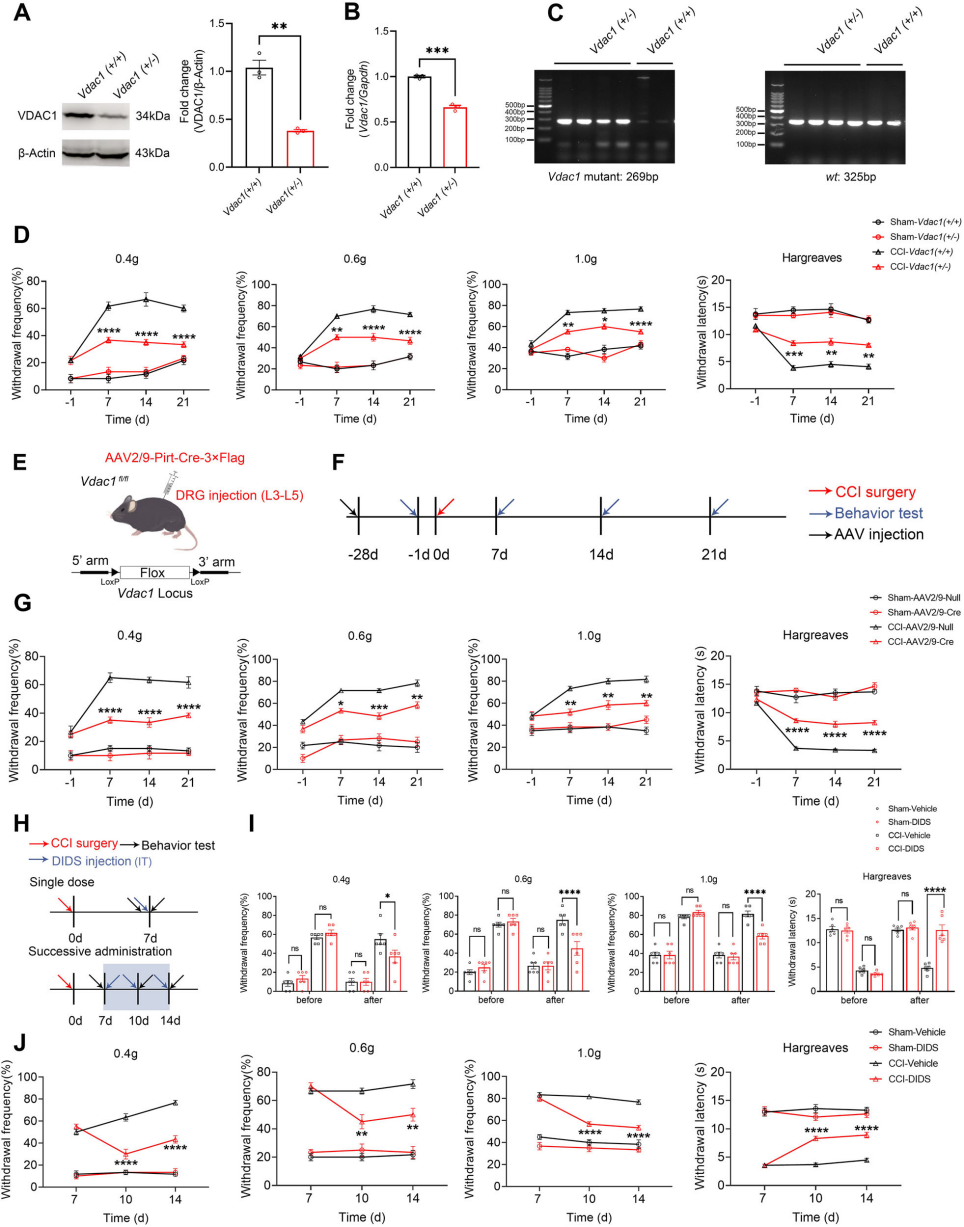
(A) Western blot analysis of VDAC1 expression in L3‐L5 DRGs from *Vdac1 (+/+)* and *Vdac1 (±)* mice. ^**^p < 0.01. Student's *t*‐test. n = 3 mice/group. (B) qPCR analyzed the mRNA level of *Vdac1* in L3‐L5 DRGs from *Vdac1 (+/+)* and *Vdac1 (±)* mice. ^***^p < 0.001. Student's *t*‐test. n = 3 mice/group. (C) PCR genetic identification results of *Vdac1 (+/+)* and *Vdac1 (±)* mice. (D) Paw withdrawal frequency to 0.4 g, 0.6 g, and 1.0 g von Frey filaments and withdrawal latency in the Hargreaves test of *Vdac1 (+/+)* and *Vdac1 (±)* mice after Sham or CCI surgery. ^*^p < 0.05, ^**^p < 0.01, ^***^p < 0.001, ^****^p < 0.0001. Three‐way ANOVA test followed by Sidak's post hoc test. n = 6 mice/group. (E) The schematic diagram of AAV‐Cre or AAV‐Null DRG injection into *Vdac1^fl/fl^* mice. (F) The schematic diagram of AAV‐Cre or AAV‐Null DRG injection and the workflow of CCI surgery and behavioral test. (G) Paw withdrawal frequency to 0.4, 0.6, and 1.0 g von Frey filaments and withdrawal latency in the Hargreaves test of mice from the AAV‐Cre or AAV‐Null group after Sham or CCI surgery. ^*^p < 0.05, ^**^p < 0.01, ^***^p < 0.001, ^****^p < 0.0001. Three‐way ANOVA test followed by Sidak's post hoc test. n = 6 mice/group. (H) The workflow of CCI surgery, DIDS injection, and behavior test. (I) Paw withdrawal frequency to 0.4, 0.6, and 1.0 g von Frey filaments and withdrawal latency in the Hargreaves test of Sham and CCI mouse models after Vehicle or DIDS single‐dose administration. n.s. not significant, ^*^p < 0.05, ^****^p < 0.0001. Three‐way ANOVA test followed by Sidak's post hoc test. n = 6 mice/group. (J) Paw withdrawal frequency to 0.4, 0.6, and 1.0 g von Frey filaments and withdrawal latency in the Hargreaves test of Sham and CCI mouse models after Vehicle or DIDS successive administration. ^**^p < 0.01, ^****^p < 0.0001. Three‐way ANOVA test followed by Sidak's post hoc test. n = 6 mice/group.

Behavior tests indicated that CCI‐induced mechanical allodynia and heat hyperalgesia were significantly reduced in *Vdac1(±)* mice, whereas the mice exhibited a normal basal pain sensation (Figure 4D). To confirm the contribution of sensory neuronal VDAC1, we injected AAV2/9‐Cre or AAV2/9‐Null into *Vdac1^fl/fl^* mice ipsilateral L3‐L5 DRGs 4 weeks before CCI surgery (Figure 4E,F). Injection of AAV2/9‐Cre, but not AAV2/9‐Null, alleviated CCI‐induced mechanical allodynia and heat hyperalgesia (Figure 4G). We next evaluated the analgesic effect of pharmacological VDAC1 inhibition. Both single‐dose and repeated intrathecal administration of DIDS markedly attenuated CCI‐induced pain behaviors (Figure 4H–J). In contrast, knockdown of *Vdac2* or *Vdac3* had no effect on neuropathic pain induced by CCI (Figure S8A–J). Together, these findings suggest that VDAC1 plays an important role in the pathogenesis of neuropathic pain.

### 2.4 VDAC1 Mediates Sensory Neuron Hyperexcitability Induced by Nerve Injury

Cellular excitability is tightly linked to changes in intracellular calcium concentration ([Ca^2+^]_i_), which also plays a critical role in the development of neuropathic pain. To examine calcium dynamics, we performed calcium imaging on DRG neurons from *Vdac1(+/+)* and *Vdac1(±)* mice after Fura‐2 AM incubation. Local application of an extracellular solution containing 30 mmol L^−1^ K^+^ to the soma was used as a depolarizing stimulus to induce neuronal depolarization, which elicited a robust increase in [Ca^2^^+^]_i_, as reflected by the rise in the fluorescence ratio (R_340/380_) [34, 35]. At the end of stimulation, 50 mmol L^−1^ K^+^ was applied at the end of recording to assess cell viability. Notably, DRG neurons from *Vdac1(+/+)* mice exhibited greater sensitivity to depolarization compared with those from *Vdac1(±)* mice, as evidenced by a significantly higher R_340/380_ ratio and a larger proportion of responsive neurons (Figure 5A–D).

**FIGURE 5:**
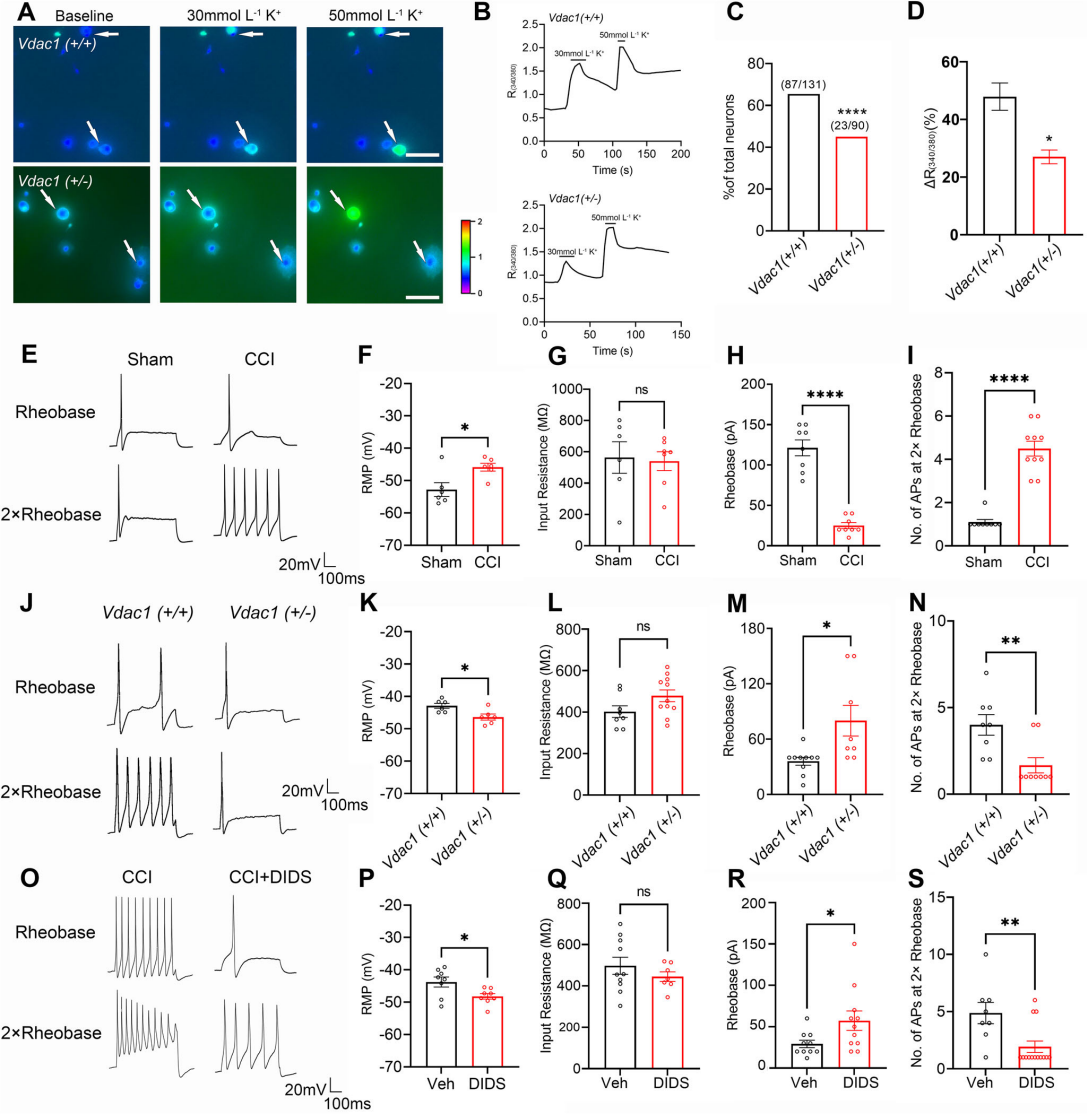
VDAC1 participates in DRG neuronal hyperexcitability in neuropathic pain conditions. (A) Representative fluorescent view and Fura‐2 ratiometric imaging of dissociated *Vdac1(+/+)* and *Vdac1(±)* neurons from CCI mice ipsilateral L3‐L5 DRGs. Scale bars: 50 µm. (B) Representative traces of calcium responses induced by KCl (30 mmol L^−1^, 50 mmol L^−1^, 15 s) in DRG neurons from *Vdac1(+/+)* and *Vdac1(±)* CCI mice. (C‐D) Quantitative analysis of the percentage of KCl‐responsive (30 mmol L^−1^) DRG neurons and the mean magnitude of KCl‐evoked (30 mmol L^−1^) calcium response from *Vdac1(+/+)* and *Vdac1(±)* CCI mice. ^*^p < 0.05, ^****^p < 0.0001. Chi‐square test (C) and Student's *t*‐test (D). (E‐I) RMP, input resistance, rheobase and number of APs at 2×rheobase of isolated neurons from Sham and CCI mice L3‐L5 DRGs. n.s. not significant, ^*^p < 0.05, ^****^p < 0.0001. Student's *t*‐test. n = 6‐10 neurons/group. (J‐N) RMP, input resistance, rheobase and number of APs at 2×rheobase of isolated‐neurons from *Vdac1(+/+)* and *Vdac1(±)* CCI mice L3‐L5 DRGs. n.s. not significant, ^*^p < 0.05, ^**^p < 0.01. Student's *t*‐test. n = 6‐10 neurons/group. (O‐S) RMP, input resistance, rheobase, and number of APs at 2×rheobase of isolated neurons from CCI mice L3‐L5 DRGs after DIDS incubation for 1 h. n.s. not significant, ^*^p < 0.05, ^**^p < 0.01. Student's *t*‐test. n = 7–11 neurons/group.

Nociceptive neuronal hyperexcitability is a hallmark of neuropathic pain. To assess this, we recorded evoked APs from small‐diameter (< 25 µm) DRG neurons of wild‐type mice under both physiological and neuropathic conditions. Patch‐clamp recordings revealed a significant hyperexcitability of sensory neurons from CCI 21 d mice DRG tissues compared to the Sham group after step current injections, as evidenced by reduced resting membrane potential (RMP), decreased rheobase, and increased numbers of APs elicited by suprathreshold stimulation (Figure 5E–I). We next compared the excitability of DRG neurons between *Vdac1(+/+)* and *Vdac1(±)* mice. In *Vdac1(±)* neurons, the CCI‐induced increase in AP firing was absent, suggesting a protective effect of partial VDAC1 deletion against neuronal hyperexcitability (Figure 5J–N). Furthermore, pharmacological inhibition of VDAC1 by DIDS incubation markedly reduced AP firing at 2× threshold current intensity compared with control neurons (Figure 5O–S). Together, these electrophysiological findings support the conclusion that VDAC1 participates in enhancing excitability of DRG sensory neurons under neuropathic pain conditions.

### 2.5 VDAC1‐Mediated ATP Transduction Contributes to Sensory Neuron Hyperexcitability

Given that VDAC1 mediates ATP transport across the mitochondrial outer membrane, its upregulation may increase cytosolic ATP availability, thereby supplying the energy required to sustain ion fluxes and repetitive action potential firing. We therefore examined the impact of VDAC1 on intracellular ATP levels in DRG neurons. VDAC1 is a highly conserved protein localized to the OMM. Our ultra‐high resolution immunofluorescence staining showed that VDAC1 was co‐localized with TOMM20, a well‐established OMM marker (Figure 6A). Considering the critical role of ATP in neuropathic pain, we next measured ATP levels under physiological and neuropathic pain conditions using LC‐MS. To normalize potential variability in nucleotide background across samples, we used the ratio of [ATP]/[adenosine diphosphate (ADP)] as an indicator of relative ATP concentration. The results revealed a significant increase in ATP levels in the L3–L5 DRGs following CCI. In contrast, ATP levels were markedly reduced in both *Vdac1(±)* mice and *Vdac1* CKO mice after CCI surgery compared to the control groups (Figure 6B–D).

**FIGURE 6:**
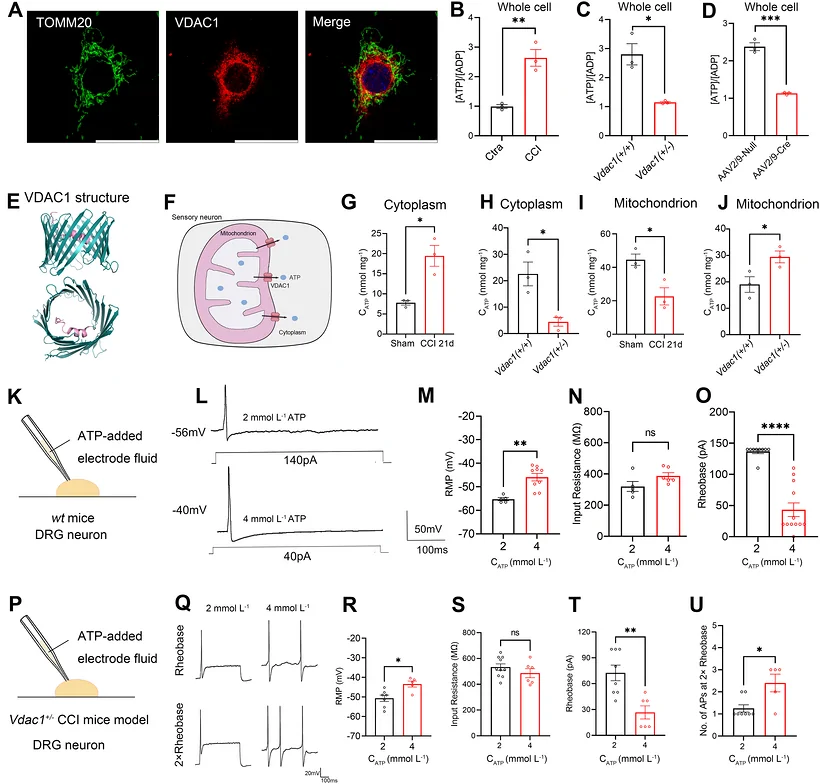
Sensory neuron activation by increased cellular ATP is VDAC1‐dependent. (A) Representative images of ultra‐high‐resolution immunofluorescence staining to co‐localize TOMM20 (green) with VDAC1 (red). Scale bar: 10 µm. (B) The ratio of [ATP] to [ADP] in the ipsilateral L3‐L5 DRGs from Sham and CCI group mice. ^**^p < 0.01. Student's *t*‐test. n = 3 mice/group. (C) The ratio of [ATP] to [ADP] in the ipsilateral L3‐L5 DRGs from *Vdac1(+/+)* and *Vdac1(±)* CCI mice. ^*^p < 0.05. Student's *t*‐test. n = 3 mice/group. (D) The ratio of [ATP] to [ADP] in the ipsilateral L3‐L5 DRGs from *Vdac1* CKO mice after CCI surgery. ^***^p < 0.001. Student's *t*‐test. n = 3 mice/group. (E) 3D structure of mouse VDAC1 (side view and top view) solved using a combined NMR/X‐ray approach (PDB code: 3EMN). (F) The schematic diagram of ATP crossing mitochondria through VDAC1. (G) The ATP concentration in the ipsilateral L3‐L5 DRGs neuronal cytoplasm from Sham and CCI group *wt* mice. ^*^p < 0.05. Student's *t*‐test. n = 3 mice/group. (H) The ATP concentration in the ipsilateral L3‐L5 DRGs neuronal cytoplasm from *Vdac1(+/+)* and *Vdac1(±)* CCI 21 d mice. ^*^p < 0.05. Student's *t*‐test. n = 3 mice/group. (I) The ATP concentration in the ipsilateral L3‐L5 DRGs neuronal mitochondria from the Sham and CCI group of *wt* mice. ^*^p < 0.05. Student's *t*‐test. n = 3 mice/group. (J) The ATP concentration in the ipsilateral L3‐L5 DRGs neuronal mitochondrion from *Vdac1(+/+)* and *Vdac1(±)* CCI 21 d mice. ^*^p < 0.05. Student's *t*‐test. n = 3 mice/group. (K) The schematic diagram of ATP addition into electrode fluid directly and neuronal single‐cell patch‐clamp of DRG neurons from naïve *wt* mice. (L) AP of DRG neurons from naïve *wt* mice after applying electrode fluid with different ATP concentrations. (M–O) RMP, input resistance, and rheobase of DRG neurons from naïve *wt* mice after applying electrode fluid with different ATP concentrations. n.s. not significant, ^**^p < 0.01, ^****^p < 0.0001. Student's *t*‐test. n = 5–11 neurons/group. (P) The schematic diagram of single‐cell patch‐clamp recording of DRG neurons from *Vdac1(±)* CCI 21 d mice after adding ATP into electrode fluid directly. (Q) APof DRG neurons from *Vdac1(±)* CCI 21 d mice after applying electrode fluid with different ATP concentrations. (R‐U) RMP, input resistance, rheobase of DRG neurons from *Vdac1(±)* CCI 21 d mice after applying electrode fluid with different ATP concentrations. n.s. not significant, ^*^p < 0.05, ^**^p < 0.01. Student's *t*‐test. n = 5–10 neurons/group.

Since VDAC1 has been implicated in regulating ATP permeability between mitochondria and the cytoplasm (Figure 6E,F), we next isolated neuronal mitochondria and quantified ATP concentrations in both the mitochondrial and cytoplasmic fractions using an ATP assay kit. In wild‐type mice, cytoplasmic ATP levels were significantly elevated following CCI. However, this increase was absent in *Vdac1(±)* mice, indicating that reduced VDAC1 expression compromises mitochondrial ATP export (Figure 6G,H). By contrast, mitochondrial ATP levels in wild‐type sensory neurons were reduced after CCI (Figure 6I). Notably, mitochondrial ATP levels were elevated in *Vdac1(±)* mice under neuropathic pain conditions, further supporting that loss of VDAC1 impairs mitochondrial ATP release into the cytoplasm (Figure 6J). These findings suggest that VDAC1‐depedent ATP efflux seems like a key mechanism coupling mitochondrial metabolism to enhanced neuronal excitability during neuropathic pain.

To directly test this link, we next investigated the functional role of elevated intracellular ATP in regulating neuronal excitability. Patch‐clamp recordings were performed while artificially increasing intracellular ATP to mimic its elevation observed under neuropathic pain conditions (Figure 6K). As expected, this manipulation induced a marked depolarization of the RMP, indicating that neurons were more prone to excitation from the resting state (Figure 6L,M). Consistently, the rheobase was also significantly reduced under high‐ATP conditions, further supporting enhanced neuronal excitability (Figure 6N,O). We further supplied excessive ATP into DRG neurons from *Vdac1(±)* CCI 21 d mice (Figure 6P). Patch‐clamp results suggested supplementation of ATP into the cytoplasm successfully restored the excitability that was reduced due to *Vdac1* knockdown, indicating the key influence of ATP efflux mediated by VDAC1 on neuronal excitability (Figure 6Q‐U 6). Together, these findings demonstrate that elevated cytoplasmic ATP directly mediates neuronal hyperexcitability, providing a mechanistic basis for the increased excitability observed in DRG neurons after CCI.

Activated neurons have been reported to exhibit elevated metabolic activity [36]. As a gatekeeper of metabolite flux between mitochondria and the cytoplasm, VDAC1 may play a role in this process. To investigate whether VDAC1 contributes to metabolic changes in DRG neurons under pain conditions, we mimicked neuronal activation by treating dissociated DRG neurons with Cap and assessed MMP using JC‐1 staining. Cap stimulation induced a significant increase in MMP, consistent with enhanced mitochondrial function and an elevated metabolic state. Notably, this increase was attenuated by *Vdac1* knockdown, suggesting that VDAC1 facilitates metabolic upregulation during nociceptive activation (Figures S9A and S10A–D). Moreover, VDAC1 expression remained unchanged in *Vdac1(±)* mice after CCI, indicating that its overexpression in neuropathic pain represents a stable adaptive change rather than a stress‐induced upregulation (Figure S9B).

## 3 Discussion

Sensory neurons operate under high energy demands during pain states to sustain processes like AP propagation, ion pumping, and synaptic transmission [37]. In this study, we identify a marked elevation in intracellular ATP levels that is dependent on nociceptive neuronal VDAC1, whose expression is markedly upregulated under neuropathic pain conditions. Electrophysiological results demonstrate that increased cytoplasmic ATP significantly enhances neuronal excitability, as indicated by changes in RMP and rheobase. Together, these findings indicate that the dynamic redistribution of ATP between mitochondria and cytoplasm through VDAC1 is critical for sustaining neuronal excitability and may actively contribute to the development and persistence of pain.

Among the three VDAC isoforms, VDAC1 showed the strongest specificity for pain‐related sensory processing. In contrast to the broader distribution of VDAC2 and VDAC3 across sensory ganglion cell types, VDAC1 was preferentially enriched in nociceptive neurons and emerged as the only subtype consistently linked to neuronal hyperexcitability and pain behavior in our study. We found that both *Vdac1* mRNA and VDAC1 protein were significantly upregulated under neuropathic pain conditions. Genetic knockdown or conditional knockout of *Vdac1* in DRG sensory neurons significantly reduced neuronal excitability and alleviated pain behaviors, indicating the critical promoting role of VDAC1 overexpression in neuropathic pain. In addition to the electrophysiological changes, *Vdac1* knockdown also attenuated the exaggerated intracellular calcium response to depolarizing stimulation, further supporting a role for VDAC1 in regulating neuronal excitability. Another potential explanation for the altered calcium responses observed after *Vdac1* loss is a change in mitochondrial calcium buffering capacity. Mitochondria participate in intracellular Ca^2^^+^ homeostasis regulation, and VDAC1 contributes to calcium transfer and signaling between the cytosol and mitochondria. Reduced depolarization‐evoked calcium signals in *Vdac1*‐deficient neurons may partly reflect altered mitochondrial handling of Ca^2^^+^ rather than a simple reduction in plasma membrane calcium entry [38]. Nevertheless, depolarization‐evoked calcium responsiveness is an important component of neuronal excitability. Together with our electrophysiological findings, these results support the conclusion that VDAC1 modulates sensory neuronal excitability. Future studies using more direct measurements of mitochondrial Ca^2^^+^ dynamics will be needed to define this mechanism more precisely.

It is worth noting that the fold change of *Vdac1* mRNA was modest, whereas it is still biologically meaningful. As a highly abundant gatekeeper of metabolite exchange in the OMM, even moderate transcriptional upregulation of *Vdac1* has been associated with mitochondrial dysfunction in previous disease models [32, 39, 40, 41]. In nervous systems, relatively small changes in the expression of excitability‐related proteins can produce substantial functional consequences [42]. In our study, the increase in *Vdac1* mRNA was accompanied by a concordant increase in VDAC1 protein, and was further supported by the changes in ATP transport, neuronal excitability, and pain behaviors. Complementary analysis of a public DRG scRNA‐seq dataset further showed that *Vdac1* is detectable across nociceptor‐ and injury‐associated neuronal populations, although this dataset did not reveal a consistent within‐cluster increase in *Vdac1* transcript levels after CCI, likely reflecting the limited sensitivity of scRNA‐seq for detecting modest changes in broadly expressed mitochondrial genes. In addition, VDAC1 overexpression was not observed in *Vdac1(±)* mice after CCI surgery, further suggesting that VDAC1 upregulation represents a stable adaptive response, rather than a stress‐induced change. However, genetic knockdown of *Vdac1* in naïve mice does not influence mitochondrial basal status (density, MMP, and MitoROX). Besides, our qPCR and western blot analyses showed that *Vdac2* and *Vdac3* gene expression were not compensatively upregulated in *Vdac*1 deficient DRG tissues. These findings suggest that the altered ATP phenotype is unlikely to result from gross mitochondrial dysfunction or isoform compensation, and instead support the interpretation that VDAC1 regulates neuronal ATP homeostasis mainly through controlling mitochondrial permeability and ATP transport.

Intracellular ATP levels have been reported to rise under pain conditions, particularly in TRPV1^+^ neurons within the spinal dorsal horn, where they contribute to neuronal hyperexcitability [43, 44]. The mechanisms by which ATP modulates neuronal hyperexcitability may involve changes in the states of ion channels and transporters [45, 46, 47]. Previous studies have shown that increased intracellular ATP enhances TRPV1 channel activity following Cap stimulation [48, 49, 50]. In addition, ATP‐sensitive potassium (K_ATP_) channels, which are widely expressed in sensory neurons, contribute to membrane repolarization. Intracellular ATP binds to the Kir6.2 subunit of these channels, promoting their closure and thereby reducing their activity [8]. In the context of neuropathic pain, elevated intracellular ATP may lead to prolonged K_ATP_ channel closure, impairing membrane repolarization and further sustaining the hyperexcitability of nociceptive neurons [47, 51]. This dysregulation of energy‐dependent ion channel function may perpetuate a vicious cycle of increased ATP production and exacerbated sensory neuron hyperactivity [52]. Moreover, ATP is also essential for the activity of Na^+^/K^+^ ATPase and Na^+^/K^+^‐Ca^2+^ (NCKX) exchangers, both of which are fundamental for maintaining ionic homeostasis [53]. However, we did not directly assess ion channel function in the present study, which remains an important direction for future investigation.

Although the present study focuses on the peripheral sensory neurons, the role of VDAC1 in pain is likely not restricted to the periphery. In the central nervous system, previous studies have shown that VDAC1 is also expressed in spinal microglia and contributes to pain maintenance under conditions such as sleep deprivation, suggesting that VDAC1 may regulate pain through glial activation and inflammatory signaling at the spinal level [18]. However, direct evidence for a pain‐related role of VDAC1 in supraspinal regions is still limited [54]. Taken together, these findings suggest that VDAC1 may participate in a continuous pain‐processing pathway that links peripheral nociceptor sensitization to central dorsal horn modulation, thereby providing a potential point of convergence between neuronal and glial mechanisms of pain maintenance. Besides, the projection of peripheral nociceptors to the spinal dorsal horn is critical for ascending nociceptive transmission and the integration of descending central modulation. The anatomical distribution of peripheral VDAC1‐positive afferents is likely to be functionally important. In the present study, we found that VDAC1 is mainly enriched in peptidergic, non‐peptidergic, and TRPV1^+^ nociceptors. Previous studies have shown that peptidergic/TRPV1‐related nociceptive afferents terminate mainly in lamina I and outer lamina II of the dorsal horn, whereas non‐peptidergic IB4^+^ fibers project preferentially to inner lamina II [55]. In addition, the central terminals of Nav1.8‐lineage nociceptors are most densely distributed in lamina I and dorsal lamina II and, after nerve injury, these nociceptors can form enhanced functional connections with lamina I spinothalamic tract neurons [56]. Based on these findings, VDAC1‐positive primary afferents are likely to project mainly to the superficial dorsal horn, especially laminae I–II, where they may influence both projection neurons and local interneurons. Although the present study does not trace the exact projection of VDAC1^+^ primary nociceptors in DRG to the spinal dorsal horn, it provides an important anatomical framework for understanding how peripheral VDAC1‐dependent changes may shape spinal pain processing.

As a mitochondrial channel, VDAC1 is critical for cell survival and metabolic regulation. In other neurological disorder conditions, VDAC1 has been implicated in several disease‐related processes, including abnormal metabolite exchange, Ca^2^^+^ dysregulation, oxidative stress, and cell death signaling [41, 57]. Its overexpression, together with excessive opening of the mitochondrial permeability transition pore (mPTP), can disturb intracellular Ca^2^^+^ homeostasis and promote apoptosis, thereby contributing to neuronal injury [9, 58]. In Parkinson's disease (PD) and other neurodegenerative conditions, VDAC1 has been linked to α‐synuclein‐related mitochondrial dysfunction and to abnormal endoplasmic reticulum–mitochondria coupling through the inositol 1,4,5‐trisphosphate receptor (IP3R)‐glucose‐regulated protein 75 (GRP75)‐VDAC1 complex, which in turn affects stress kinase signaling, inflammatory responses, and neuronal survival [12, 59, 60]. Importantly, some neurological disorders in which VDAC1 dysfunction has been reported, such as PD, Alzheimer's disease (AD), and amyotrophic lateral sclerosis (ALS), are also frequently accompanied by chronic pain symptoms [61, 62, 63]. On the other hand, mitochondrial stress, oxidative injury, neuroinflammation, and maladaptive calcium signaling have also been reported to participate in pain maintenance. Mitochondrial injury is a prominent feature of trigeminal neuropathic pain; restoring mitochondrial function by boosting nicotinamide adenine dinucleotide (NAD^+^) can alleviate pain hypersensitivity and related pathological changes [64]. These observations raise the possibility that a similar pattern of VDAC1 dysregulation may lead to different clinical manifestations depending on the neuronal population or anatomical level involved. It is possible that chronic pain and certain neurodegenerative diseases may share common VDAC1‐related upstream pathophysiological mechanisms and may even show partial common comorbidity. However, these possibilities were not directly addressed in the present study and might require further investigation.

From a clinical perspective, the present findings suggest that VDAC1 is not only a single molecular target, but also an entry point into a broader metabolic strategy for pain treatment. By linking VDAC1‐dependent mitochondrial ATP redistribution to nociceptor hyperexcitability, our study points to mitochondrial metabolite handling as a therapeutic concept distinct from conventional ion channel‐ or inflammation‐centered approaches. However, several limitations should be noted. First, although Pirt‐Cre preferentially targets small‐ to medium‐diameter sensory neurons and our data support a major contribution from nociceptive populations, it remains a broad DRG sensory neuron driver rather than a strictly nociceptor‐specific tool; future studies using subtype‐restricted Cre lines will be required to define the contribution of VDAC1 in distinct sensory neuron populations [22, 23, 24]. Second, although genetic and pharmacological data support a role for VDAC1 in mitochondrial ATP efflux, DIDS is a broad‐spectrum anion transport inhibitor. It might also act on multiple plasma membrane anion exchangers and chloride (Cl^−^) channels, including DIDS‐sensitive solute carrier family 4 (SLC4) family members, volume‐regulated anion channels (VRAC), and Ca^2^^+^‐activated Cl^−^ channels, some of which have independent roles in DRG excitability and pain signaling [65, 66]. Thus, its analgesic effects should therefore be interpreted as supportive rather than VDAC1‐specific evidence; more selective pharmacological or genetic tools will be needed to directly link VDAC1‐mediated ATP transport to neuronal hyperexcitability [65, 66]. Third, although intrathecal delivery reduces the confounding influence of systemic exposure and is widely used for DRG drug administration, it still does not allow a clear distinction between DRG‐mediated and spinal cord‐mediated effects, and more localized approaches will be needed in future studies. Finally, our bioinformatic analyses suggested that *Vdac1* upregulation after nerve injury may be associated with injury‐related transcription factors and mitochondrial metabolic pathways. Besides, post‐translational modification (PTM) might also contribute to VDAC1 upregulation. Previous studies have reported several VDAC1 PTMs, including phosphorylation, acetylation, and oxidative modification, which may influence channel conductance, protein interactions, or mitochondrial permeability [67]. However, these mechanisms were only predicted in the present study and require further experimental validation in future work.

## 4 Conclusion

Our study demonstrates that VDAC1 plays a crucial role in regulating ATP transport within nociceptive neurons and highlights its significant contribution to neuronal excitability and neuropathic pain transmission. Targeting VDAC1 may provide a more precise and effective approach to neuropathic pain relief.

## 5 Materials and Methods

### 5.1 Animals

Male mice born after 4 weeks (18–20 g) were used in this study. Mice were housed under a controlled environment (23 ± 3°C, 12‐h light/12‐h dark cycle) with random access to a standard diet and water. All experiments were approved by the Institutional Animal Care and Use Committee in the Chinese Academy of Medical Sciences, Institute of Basic Medical Sciences (approval number: #211‐2014). Mice were randomly assigned to experimental groups.

*Vdac1^fl/fl^* and *Vdac1(±)* mice were purchased from GemPharmatech LLC. (Jiangsu, China). *Vdac1(±)* mice were generated by crossing *Vdac1(+/+)* mice with *wt* mice to generate the heterozygous *Vdac1(±)* mice. The *Vdac1(+/+)* mice were selected to be littermates.

### 5.2 Human Sample Sources

The trigeminal ganglion (TG) and dorsal root ganglion (DRG) samples were recruited from the National Human Brain Bank for Development and Function, Chinese Academy of Medical Sciences, and Peking Union Medical College (PUMC) in Beijing, China. The research protocol was approved by the Institutional Review Board of the Institute of Basic Medical Sciences of the Chinese Academy of Medical Sciences, PUMC, Beijing, China (approval numbers: 009–2014, 031–2017, and 2022125). For both human DRG (L4) and TG, tissues from the left and right sides were collected for immunostaining, and the TG samples were taken from the ganglion proper containing neuronal cell bodies.

### 5.3 Drugs and Administration

Cap (100 mmol) was dissolved in EtOH as a storage solution at −20°C. For the behavior test, Cap was prepared in PBS and was subcutaneously injected into the plantar hind paw by insulin syringe (10 µL/mice). For cellular stimulation, Cap was prepared with the complete cell culture medium. The anion channel inhibitor 4,4'‐Diisothiocyanostilbene‐2,2'‐disulfonic acid (DIDS) was first dissolved in dimethyl sulfoxide (DMSO) and diluted in PBS for intrathecal injection (2 mg kg^−1^, 0.04 mg in 10 µL/mice) [27]. The behavior test was performed 1 h after drug administration. Small interfering RNA (siRNA) targeting *Vdac1*, *Vdac2*, *Vdac3* (*siVdac1*, *siVdac2*, and *siVdac3*, 50 nmol, KeyGen Biotech, Jiangsu, China) and scramble RNA (scRNA, 50 nmol L^−1^, KeyGen Biotech, Jiangsu, China) were dissolved in PBS solution prepared with RNase‐free water (0.5 µg µL^−1^), and were injected into DRG for continuous 3 days before behavior assay.

### 5.4 Intra‐DRG Injection

Intra‐DRG injection was performed as previously described with the minor adjustments [68]. Briefly, mice were anesthetized with sodium pentobarbital (40 mg kg^−1^), and a small dorsolateral laminectomy was performed to expose L3‐L5 DRGs. rAAV or scRNA/siRNAs were injected into the DRG using a 30‐G syringe. After injection, the syringe was held for 30 s to prevent leakage.

### 5.5 Intrathecal Injection

Intrathecal injection was performed using an insulin syringe to deliver the drugs into the subarachnoid space between the L4 and L5 spinal levels. The instant tail‐flick reaction induced by the needle entry was regarded as valid a proper depth of the needle depth. After injection, the syringe was held for 10 s to prevent leakage.

### 5.6 Intradermal Injection

Intradermal injection was performed under short‐term anesthesia with isoflurane. Cap (0.05 mmol L^−1^, 10 µL/mice) was subcutaneously injected into the plantar hind paw by insulin syringe. After successful injection, obvious skin mounds formed on the surface of the hind feet of mice. After injection, the syringe was held for 30 s to prevent leakage.

### 5.7 Chronic Constriction Injury (CCI) Surgery

The CCI surgery was directed by the study of Bennett and Xie [69]. Mice were completely anesthetized with isoflurane. After the sciatic nerve on the right side was exposed at the mid‐thigh level, three nug ligatures of non‐absorbent sutures (6‐0) were loosely tied around the nerve with about 0.5 mm space between the knots. The sciatic nerves of sham mice were exposed without ligation. The mice were closely monitored after the operation until they regained consciousness. Mice in the Sham group underwent the same surgical procedure, including incision of the ipsilateral hindlimb skin and muscle separation, but without sciatic nerve ligation.

### 5.8 Pain Behavioral Tests

#### 5.8.1 Von Frey Test

To be fully acclimated, mice were placed in the test chamber on the metal mesh 30 min before each behavioral assay. Mechanical allodynia was assessed by applying incremental von Frey filaments (0.40, 0.60, and 1.00 g) to the plantar surface at a vertical angle for 3 s. Each mouse was tested 10 times, and the percentage of paw withdrawal response was calculated.

#### 5.8.2 Hargreaves Test

For thermal hyperalgesia, the Hargreaves test was applied with the radiant heat source of a thermal stimulator (BME‐410C Plantar Test Apparatus, China). The radiation source was focused on the hindpaw for 3 times, and the average withdrawal latency was calculated as withdrawal latency. The timeline of the surgery, drug delivery, and behavior test of each experiment was shown by a diagram in the relative figures.

### 5.9 Primary Dorsal Root Ganglion Neurons Culture

L3‐L5 DRGs were harvested and transferred into oxygenated artificially formulated extracellular fluid for cleaning and mixing, which contained (in mmol L^−1^): NaCl 145, KCl 3, CaCl_2_·2H_2_O 2, MgCl_2_·6H_2_O 2, HEPES 10, Glucose 10, with an osmolarity of 290–300 mOsm and a pH that was adjusted to 7.2–7.4 using NaOH. The DRGs were then digested with Liberase TM (Roche, Basel, Switzerland) for 20 min and for another 15 min with Liberase TL (Roche, Basel, Switzerland) and papain (30 U mL^−1^; Worthington Biochemical, Lakewood, NJ, USA) in extracellular fluid at 37°C. The tissue was blown gently after enzymatic digestion with a fire‐polished Pasteur pipette. After abstersion with a culture medium containing 0.5 mg mL^−1^ bovine serum albumin (Sigma‐Aldrich, Saint Louis, USA) and 0.5 mg mL^−1^ trypsin inhibitor (Roche, Basel, Switzerland), the cells were placed on poly‐D‐lysine/laminin‐coated circular glass coverslips (8 mm for patch‐clamp, and 12 mm for calcium imaging). The culture medium contained equal amounts of DMEM and F12 with 10% FBS, 1% glutamine, and 1% penicillin/streptomycin. The cells were maintained at 37°C in a humidified atmosphere of 95% O_2_ and 5% CO_2_ and were used within 24 h.

To identify the influence of VDAC1 on mitochondrial membrane potential (MMP), *siVdac1* or scRNA was transfected for 24 h before Cap stimulation. To pharmacologically block VDAC1, DIDS (100 µmol L^−1^; Sigma–Aldrich, USA) or DMSO was added to the culture medium 1 h before patch‐clamp recording.

### 5.10 Patch‐Clamp Recording

Mice (6‐8 weeks old, male, C57BL/6 wild type or *Vdac1* heterozygote) L3‐L5 DRGs were isolated. The cells were then perfused with artificially formulated extracellular fluid saturated with 95% O_2_ and 5% CO_2_. The patch pipettes were pulled from borosilicate glass capillaries with filaments using a flaming micropipette puller (P‐100, Sutter Instruments, USA). The initial resistances of patch pipettes were about 3–5 MΩ when filled with the internal pipette solution, which contained (in mmol L^−1^): K^+^‐gluconate 120, KCl 30, MgCl_2_·6H_2_O 2, HEPES 10, Mg‐ATP 2, CaCl_2_·2H_2_O 1, EGTA 11, with an osmolarity of 290–300 mOsm and a pH that was adjusted to 7.2–7.4 using Tris base. For tests observing ATP influence on neuronal excitability, the internal pipette solution contained 4 mmol Mg‐ATP. Whole‐cell current recordings were performed at room temperature (20–22°C) using a Multiclamp 700B amplifier with pClamp 11 software (Molecular Devices). Small‐diameter (≤ 25 µm) neurons were chosen, and the neurons were recorded when their resting membrane potential (RMP) was more negative than −40 mV. Action potentials (APs) were evoked by a series of depolarizing current steps (duration: 500 ms, 50 pA‐1 nA).

### 5.11 Intracellular Calcium Imaging

Calcium imaging was performed on the small‐diameter (≤ 25 µm) DRG neurons as described previously [70]. Briefly, DRG neurons were loaded with Fura 2‐acetoxymethyl ester (2 µmol L^−1^ in DMSO; Invitrogen, USA) in the dark in an incubator for 45 min. After being washed 3 times with sterile PBS, DRG neurons were placed in a recording chamber continuously perfused with extracellular fluid at a flow rate of 1.5 mL/min at room temperature. HEPES extracellular fluid containing 30 mmol L^−1^ K^+^ was applied to approximately activate neurons, and 50 mmol L^−1^ K^+^ was used to confirm the viability of neurons at the end of each experiment. All reagents were applied locally to the neuronal cell bodies through a micropipette (with a tip diameter of 100 µm) and an 8‐channel pressure‐controlled drug application system (AutoMate Scientific, Berkeley, CA, USA). The fluorescence signal was captured by a ratiometric imaging system (Nikon), and the ratio of 340/380 nm fluorescence intensity (*R_340_/R_380_*) was used as a relative measurement of intracellular calcium concentration ([Ca^2+^]_i_).

### 5.12 Polymerase Chain Reaction (PCR)

#### 5.12.1 Single Cell Quantitative PCR (qPCR)

The individual DRG neurons picked with glass pipettes were harvested in PCR tubes containing 10 µL of lysis buffer and RNase inhibitor (Invitrogen, CA, USA); the samples were flash frozen and stored at −80°C until cDNA synthesis. For the negative control, a sample of the solution without any cell contents was used. For positive control, the RNA from whole DRG lysate was used. The RNA was reverse transcribed to cDNA using SuperScript III CellsDirect cDNA Synthesis Kit (Invitrogen, CA, USA) according to the manufacturer's recommended protocol. qPCR was performed using 1.5 µL of cDNA and SYBR premix Ex Taq^TM^ (Takara, Japan) on a CFX96 Real‐Time PCR Detection System (Bio‐Rad, Hercules, CA, USA). *Gapdh* was used as an internal control. The primers were listed in Table S1.

#### 5.12.2 Tissue RNA Extraction and qPCR

Total RNAs of L3‐L5 DRGs were extracted using Trizol reagent (Invitrogen, USA). The concentration of RNA samples was quantified according to UV absorbance ratios at wavelengths of 260 and 280 nm (*A_260/280_*). The following reverse transcription by RT Master Mix (Takara, Japan) was performed according to the manufacturer's instructions. qPCR amplifications were conducted by CFX96 Real‐Time PCR Detection System with SYBR premix Ex Taq. The housekeeping gene *Gapdh* was used for normalization. The primers were listed in Table S2. Relative mRNA expression was calculated using the 2^−ΔΔCt^ method. After normalization to *Gapdh*, the mean value of the control group was set as 1, and the data from the experimental group were presented as fold change relative to the Sham group.

### 5.13 Immunofluorescence Staining

#### 5.13.1 Immunofluorescence for Cryosections

Mice were anesthetized with sodium pentobarbital (40 mg/kg) and then perfused with ice‐cold phosphate buffer saline (PBS) followed by 4% paraformaldehyde (PFA, Servicebio, Wuhan, China) through the ascending aorta. The L3‐L5 DRGs were collected, fixed in 4% paraformaldehyde for 1 h, and then dehydrated in 30% (*w/v*) sucrose for 24 h at 4°C before they were embedded and frozen in OCT compound (Tissue‐Tek, Japan). Tissues were cut to 14 µm in a cryostat for immunofluorescence staining. After being incubated with 10% horse serum for 1 h, tissue sections were incubated at 4°C overnight with the primary antibodies. After being washed with PBS, the tissues were incubated with the proper secondary antibodies or Alexa Fluor 594‐conjugated isolectin B4 (IB4) (1:200, Invitrogen, USA) for 1 h at room temperature. The slides were cover‐slipped with fluorescent mounting medium (ZSGB‐BIO, ZLI‐9557) and imaged after drying. For human tissues, the slides were incubated with 0.1% Sudan Black B for 0.5 h before being coverslipped. All antibodies used for immunochemistry are listed in Table S3. The percentage of positive neurons to total neurons was calculated and statistically analyzed. Images were captured by a laser confocal microscopic imaging system (Olympus FV1000 and FluoView software, Olympus, Japan).

#### 5.13.2 Immunofluorescence for Primary DRG Neurons

Primary DRG neurons were post‐fixed in cold 4% PFA for 15 min. After being permeabilized with 0.15% Triton X‐100 for 15 min, the coverslips were incubated with 10% horse serum for 20 min. The glass coverslips were incubated with primary antibodies at 4°C overnight. After washing with PBS, the coverslips were incubated with corresponding secondary antibodies for 1 h at room temperature and mounted with ProLong Gold Antifade Mountant (Thermo Scientific, CA, USA). Images were acquired with a laser confocal microscopic imaging system (TCS‐SP8 STED 3X, Leica, Germany). All antibodies used for immunochemistry are listed in Table S3.

### 5.14 Western Blot

Mice were deeply anesthetized with sodium pentobarbital (40 mg/kg), and the L3‐L5 DRGs were collected. Tissues were homogenized on ice in RIPA buffer (CW‐bio, Beijing, China) with protease inhibitor (AbMole BioScience, Houston, TX, USA) and phosphatase inhibitor (CW‐Bio, Beijing, China). After centrifuging, supernatants were collected and denatured with 5× SDS‐PAGE loading buffer (Servicebio, Wuhan, China) for 10 min at 95°C. Equal volumes of lysates (10 µL) were separated by SDS‐polyacrylamide gels (10%) and transferred to a PVDF membrane (Sigma–Aldrich, CA, USA). After blocking with 5% BSA in TBST (CW‐bio, Beijing, China) for 1 h at room temperature, the membranes were incubated in TBST overnight at 4°C with primary antibodies. The membranes were washed with TBST and then incubated with secondary antibodies diluted with TBST for 1 h at room temperature. Bands were determined using an ECL Kit (Tanon, Shanghai, China). The primary and secondary antibodies used for western blot analysis are listed in Table S4.

### 5.15 Mitochondrial Membrane Potential (MMP) Measurement

DRG neurons were washed by pre‐cooling sterile PBS 3 times after scRNA or *siVdac1* transfection. Cap (2 µmol) was added to complete the cell culture medium to stimulate the neurons for 30 min. Then the strength of MMP was evaluated using the Mitochondrial Staining Kit (Sigma–Aldrich, Saint Louis, USA) according to the manufacturer's instructions. JC‐1 working solution was mixed with the medium in equal volume, and it was added to the 8‐well confocal cell culture dish. After 20 min incubation, the mixture was aspirated, and the cells were washed twice with growth medium. The cells were observed under the confocal microscope (TCS‐SP8 STED 3X, Leica, Germany). Depolarized mitochondria (JC‐1 monomers) emitted green fluorescence (excitation at 488 nm, emission at 499 nm). Polarized mitochondria (JC‐1 aggregates) emitted red fluorescence (excitation at 532 nm, emission at 544 nm). The ratio of red to green fluorescence intensity indicated the status of MMP in the neurons, with a decrease in the red/green ratio indicating mitochondrial depolarization. The intensity was analyzed by LAS X software (Leica, Germany) for quantification. For each experimental condition, at least 3 random fields of view were selected. Mean fluorescence intensity of all neurons was measured in each selected field.

### 5.16 MitoROX Measurement

DRG neurons were isolated from *Vdac1(+/+)* or *Vdac1(±)* mice and cultured for 3 h in an 8‐well confocal cell culture dish. Cells were washed by pre‐cooling sterile PBS 3 times before measurement. The MitoROX measurement was performed using the MitoSOX Green indicator according to the manufacturer's instructions. MitoSOX Green reagent in anhydrous DMF to make a 1 mmol stock solution. The working solution was prepared by extracellular fluid (1 µmol) and was added to the cell culture dish at a volume of 200 µmol per well. After 20 min of incubation, the mixture was aspirated, and the cells were washed twice with growth medium. The cells were observed under the confocal microscope (TCS‐SP8 STED 3X, Leica, Germany) using a standard FITC filter. The intensity was analyzed by LAS X software (Leica, Germany) for quantification. For each experimental condition, at least 3 random fields of view were selected. Mean fluorescence intensity of all neurons was measured in each selected field of view.

### 5.17 Transmission Electron Microscope (TEM)

TEM was used to measure the number of mitochondria and mitochondrial cristae in sensory neurons of *Vdac1(+/+)* or *Vdac1(±)* mice L3‐L5 DRGs. Tissues were harvested and fixed in 2.5% glutaraldehyde (EM Grade) at 4 °C overnight. After fixation in 1% osmium acid, samples were subsequently dehydrated and placed in embedding molds in a standard fashion. Ultrathin sections of 0.08 µm were stained with lead citrate and uranyl acetate. Those sections were then examined using a transmission electron microscope (JEM‐1400 Plus, JOEL, Ltd., Tokyo, Japan). Small neurons (≤ 25 µm) were selected to measure the number of mitochondrial cristae. For each experimental condition, at least 3 random fields of view were selected. The mean value of all neurons was measured in each selected field of view.

### 5.18 ATP Concentration Measurement

The concentrations of ATP and ADP in mouse DRG tissues were quantified using liquid chromatography–mass spectrometry (LC‐MS). Briefly, DRG tissues were rapidly harvested and immediately snap‐frozen in liquid nitrogen to prevent nucleotide degradation. Tissues were homogenized in ice‐cold 80% methanol (*v/v*) containing 0.1% formic acid to extract intracellular metabolites. The homogenates were centrifuged at 15 000 g for 15 min at 4 °C, and the supernatants were collected and filtered through 0.22 µm membrane filters. The flow rate was set to 0.3 mL min^−1^ with a gradient elution program optimized for nucleotide separation. Quantification of ATP and ADP was performed using standard curves generated from serial dilutions of commercially available ATP and ADP standards. Data were normalized to tissue weight and analyzed using appropriate LC‐MS software. To avoid the error caused by the background level between groups, the concentration of tissue was displayed by the ratio of [ATP] to [ADP].

For mitochondria and cytoplasm, the ATP concentration was detected by the Enhanced ATP Assay Kit (Biyotime Biotechnology, Shanghai, China) according to the manufacturer's instruction book. After extraction using the Minute Mitochondrial Isolation Kit for Mammalian Cells and Tissues (Invent Biotechnologies, Inc., Minnesota, USA), the mitochondria were lysed using sample lysis buffer. Then the samples were centrifuged at 4°C for 5 min at 12 000 g, and the supernatant was used for subsequent assays. The samples’ concentration was calculated by the ATP standard curve. ATP concentration in the DRG neuronal cytoplasm was detected by the same protocol after being mixed with the lysis buffer directly. For each group, L3‐L5 DRGs from 3 mice were detected.

### 5.19 Bioinformatic Analysis

Public DRG transcriptomic datasets related to peripheral nerve injury were analyzed to examine *Vdac1* expression patterns and explore potential upstream regulatory mechanisms. scRNA‐seq data from mouse DRG after CCI were obtained from the Gene Expression Omnibus (GEO) database (GSE216039). Processed raw count matrices were downloaded from GEO and analyzed using Python and Scanpy. Cells were filtered based on quality control metrics, including detected genes, total counts, and mitochondrial transcript fraction. After normalization and log transformation, highly variable genes were selected, followed by principal component analysis, neighborhood graph construction, UMAP visualization, and Leiden clustering.

Neuronal clusters were annotated according to established DRG neuronal marker genes and marker signatures reported in the original GSE216039 study [28]. Annotated neuronal populations included NP1, NP3, peptidergic neuronal populations, CCI‐induced injury‐associated clusters, and other DRG neuronal subtypes. *Vdac1* expression was visualized across annotated neuronal populations using UMAP feature plots, dot plots, and summary bubble plots. Comparisons between Sham and CCI conditions were performed within defined neuronal groups, with sample‐level summaries used to evaluate consistency across biological samples.

To explore transcriptional programs associated with *Vdac1* expression, candidate gene modules related to injury/AP‐1/STAT3 signaling, inflammatory TNF/IL6/NFKB/STAT3 signaling, HIF1A/NRF1‐associated mitochondrial programs, ROS/MAPK/AP‐1 signaling, and mitochondrial ATP transport were curated based on literature and pathway relevance. Module scores were calculated for each cell using a sparse‐safe expression‐bin‐matched control gene scoring method analogous to Scanpy score genes. *Vdac1*‐high and *Vdac1*‐low cells were defined based on *Vdac1* expression quantiles, and module scores were compared between these groups. Spearman correlation analysis was performed to assess associations between *Vdac1* expression and module scores across all cells and selected neuronal populations. Sensitivity analyses were performed by recalculating mitochondrial and ATP transport module scores after removing *Vdac1* or the Vdac gene family from the corresponding gene sets.

Candidate transcription factors, including Atf3, Jun, Fos, Stat3, Rela, Nfkb1, Hif1a, Nrf1, Atf1, and Smad1, were examined across annotated neuronal groups. The candidate transcription factors were cross‐referenced with their expression patterns in the scRNA‐seq dataset, and putative binding motifs in the *Vdac1* promoter region were examined using publicly available motif databases, including JASPAR.

Single‐cell preprocessing, UMAP visualization, and Leiden clustering were performed with Python and Scanpy. The locally verified Scanpy‐capable environment contained Python 3.10.19 and Scanpy 1.11.5. Downstream summaries, statistical analyses, and figure generation were performed using AnnData, NumPy, pandas, SciPy, Matplotlib, seaborn, scikit‐learn, statsmodels, and Pillow. Cell‐level statistical comparisons were considered exploratory because individual cells are not independent biological replicates. Public transcriptomic results were interpreted as supporting cellular localization and subtype‐specific transcriptional associations, while experimental assays were used as the primary evidence for VDAC1 protein‐level and functional changes.

### 5.20 Statistical Analysis

Data are presented as means with standard errors (mean ± SEM). Statistical analyses were performed using the SPSS software (version 17.0). A Student's *t*‐test was used to evaluate the statistical significance of a difference between the two groups. Chi‐square tests were used to compare between two or more events. Comparisons among multiple groups, including saline control and drug‐treated groups, were analyzed using Two‐way ANOVA followed by Sidak's post hoc test. For repeated behavioral measurements over time in Sham control and CCI groups. Three‐way ANOVA followed by Sidak's post hoc test was applied. *p<0.05* was considered statistically significant in all analyses.

## Author Contributions

Conceptualization: C.M. Funding acquisition: C.M. and T.W. Formal analysis: F.S, and N.Y. Data curation: F.S, N.Y., X.T, and Y.M. Validation: C.M., T.W., and N.Y. Project administration: C.M., T.W., F.S. and N.Y. Methodology: F.S. and N.Y. Investigation: F.S, N.Y., L.Y, X.T, and Y.M. Visualization: F.S. and N.Y. Supervision: C.M. and T.W. Resources: C.M. and T.W. Writing – original draft: F.S. and N.Y. Writing – review and editing: C.M. and T.W.

## Funding

This study was supported by grants from: STI2030‐Major Project #2021ZD0201100 Task 1 #2021ZD0201101.

## Conflicts of Interest

The authors declare no conflict of interest.

## Supporting information




**Supporting File 1**: advs76413‐sup‐0001‐SuppMat.docx.

**Supporting File 2**: advs76413‐sup‐0002‐TableS1.xlsx.

**Supporting File 3**: advs76413‐sup‐0003‐TableS2.xlsx.

**Supporting File 4**: advs76413‐sup‐0004‐TableS3.xlsx.

**Supporting File 5**: advs76413‐sup‐0005‐TableS4.xlsx.

## Data Availability

The data that supports the findings of this study are available from the corresponding author upon reasonable request. For bioinformatic analysis, scRNA‐seq of mice from Sham and CCI 7 d groups were obtained from GEO accession #GSE216039.
