# Supplementary material for: VDAC1 Upregulation Induces Hyperexcitability of Nociceptive Sensory Neurons Via Enhanced Mitochondrial Atp Efflux in Neuropathic Pain
Source: Adv Sci (Weinh). 2026 Jul 7;13(54):e23223. doi: 10.1002/advs.202523223 (PMC13339037; doi:10.1002/advs.202523223)
Supplement: Supplementary file 1 [file ADVS-13-e23223-s003.docx]

**VDAC1 upregulation induces hyperexcitability of nociceptive sensory neurons via enhanced mitochondrial ATP efflux in neuropathic pain**

Fengrun Sun^1, #^, Ning Yu^1, #^, Liang Yu^1^, Xiaofen Tian^1, 2^, Yujia Ma^1^, Yikuan Xie^1^, Tao Wang^1, *^, Chao Ma^1, 3, 4, *^

1 State Key Laboratory of Common Mechanism Research for Major Diseases, Department of Human Anatomy, Histology and Embryology, Neuroscience Center, Joint Laboratory of Anesthesia and Pain, Institute of Basic Medical Sciences Chinese Academy of Medical Sciences, School of Basic Medicine Peking Union Medical College, Beijing, China, 100005.

2 Department of Anatomy, Key Laboratory of Human Brain Bank for Functions and Diseases of Department of Education of Guizhou Province, Guizhou Medical University, Guiyang, Guizhou, China, 550009.

3 National Human Brain Bank for Development and Function, Beijing, China.

4 Chinese Institute for Brain Research, Beijing, China.

^#^ Fengrun Sun and Ning Yu contributed equally to this work.

* Corresponding author:

Tao Wang

Email: wangtao@ibms.pumc.edu.cn

Phone/Fax: +86-10-69156469

Address: No. 5 DongDanSanTiao, Dongcheng District, Beijing 100005, China

Chao Ma

Email: machao@ibms.cams.cn

Phone/Fax: +86-10-69156469

Address: No. 5 DongDanSanTiao, Dongcheng District, Beijing 100005, China

**Figure S1. VDAC subtypes’ expression in satellite cells of mice DRG and human sensory ganglia.** (A) The representative images of double staining of VDAC1/VDAC2/VDAC3 (green) with GS (red) of mice DRG. Scale bars: 50 μm. (B) The representative images of double staining of VDAC1/VDAC2/VDAC3 (green) with GS (red) of human TG. Scale bars: 50 μm. (B) The representative images of double staining of VDAC1/VDAC2/VDAC3 (green) with GS (red) of human DRG. Scale bars: 50 μm.

**Figure S2**. **VDAC1 expression in human nociceptive neurons.** (A-C) The representative images of double staining of VDAC1 (green) with CGRP, IB4, and TRPV1 (red) of human TG and DRG. Scale bars: 50 μm.

**Figure S3**. **VDAC2 and VDAC3 expression in human sensory neurons.** (A) The representative images of double staining of VDAC2 (green) with PGP9.5 (red) of human TG and DRG. Scale bars: 50 μm. (B) The representative images of double staining of VDAC3 (green) with PGP9.5 (red) of human TG and DRG. Scale bars: 50 μm.

**Figure S4.** **The conditional knock-out of *Vdac1* in DRG does not matter normal basal pain sensation of mice.** (A) The schematic diagram of AAV-Cre or AAV-Null DRG injection and behavioral test. (B-C) Western blot and immunofluorescence staining verification of the *Vdac1* knock-out efficacy. (D-G) Paw withdrawal frequency to 0.4 g, 0.6 g, and 1.0 g von Frey filaments and withdrawal latency in Hargreaves test of *Vdac1* CKO mice. n.s. not significant. Student’s t test. n=6 mice/group.

**Figure S5. Public scRNA-seq analysis of *Vdac1* expression across DRG neuronal subtypes in Sham and CCI 7 d mice.**

(A) UMAP visualization of annotated DRG neuronal populations from the public CCI scRNA-seq dataset. (B) Dot plot shows representative subtype and injury-related marker genes across selected neuronal groups. Dot size indicates the fraction of positive cells, and color indicates mean log-normalized expression. (C) UMAP visualization of target nociceptor- and injury-associated neuronal groups used for *Vdac1* analysis, including NP1, NP3, PEP1, PEP2, PEP1/2, injury-NP, and CCI-induced clusters. (D) Bubble plot shows mean log-normalized *Vdac1* expression and detection fraction across target neuronal groups. (E) Sample-level comparison of mean *Vdac1* expression between Sham and CCI conditions in selected neuronal groups. Small circles represent individual biological samples, diamonds indicate condition-level means across samples.

**Figure S6. Bioinformatic prediction of potential transcriptional programs associated with *Vdac1* expression.** (A) Dot plot shows the expression of candidate transcription factor genes across target neuronal groups. Dot size indicates the fraction of positive cells, and color indicates mean log-normalized expression. (B) Predicted Atf3, Jun, and Stat3 binding motifs associated with the *Vdac1* promoter region based on JASPAR database analysis. (C) Heatmap showing Spearman correlation between *Vdac1* expression and module scores related to injury/AP-1/STAT3, inflammatory signaling, HIF1A/NRF1-associated mitochondrial programs, ROS/MAPK/AP-1, and mitochondrial ATP transport pathways. Numbers indicate Spearman correlation coefficients.

**Figure	S7. *Vdac1* deficiency does not influence baseline of mitochondrial status.** (A-B) Representative MitoROX staining images (green) of DRG neurons from naïve *Vdac1(+/+)* and *Vdac1(+/-)* mice, along with quantitative analysis of MitoROX intensity. Scale bar: 50 μm. n.s. not significant. Student’s t test. n=3 mice/group. (C) Representative TEM images of mitochondria in the DRG neurons from naïve *Vdac1(+/+)* and *Vdac1(+/-)* mice. Scale bar: 1 μm. (D) Quantitative analysis of mitochondrial number and mitochondrial cristae number of DRG neurons from naïve *Vdac1(+/+)* and *Vdac1(+/-)* mice. n.s. not significant. Student’s t test. n=3 mice/group. (E) Representative JC-1 staining images of DRG neurons from naïve *Vdac1(+/+)* and *Vdac1(+/-)* mice. Scale bar: 50 μm. (F) Quantitative analysis of neuronal MMP accessed by the intensity ratio of JC-1 aggregates (red) to monomer (green). n.s. not significant. Student’s t test. n=3 mice/group. (G) Western blot analysis of VDAC2 expression of DRG neurons from naïve *Vdac1(+/+)* and *Vdac1(+/-)* mice. n.s. not significant. Student’s t test. n=3 mice/group. (H) qPCR analyzes the mRNA level of *Vdac2* in L3-L5 DRG neurons from naïve *Vdac1(+/+)* and *Vdac1(+/-)* mice. n.s. not significant. Student’s t test. n=3 mice/group. (I) Western blot analysis of VDAC3 expression of DRG neurons from naïve *Vdac1(+/+)* and *Vdac1(+/-)* mice. n.s. not significant. Student’s t test. n=3 mice/group. (H) qPCR analyzes the mRNA level of *Vdac3* in L3-L5 DRG neurons from naïve *Vdac1(+/+)* and *Vdac1(+/-)* mice. n.s. not significant. Student’s t test. n=3 mice/group.

**Figure S8. Genetic knock-down of *Vdac2* or *Vdac3* exhibits no effect on neuropathic pain alleviation caused by CCI surgery.** (A) The schematic diagram of scRNA or si*Vdac2* DRG injection, and the workflow of CCI surgery and behavioral test. (B-E) Paw withdrawal frequency to 0.4 g, 0.6 g, and 1.0 g von Frey filaments and withdrawal latency in Hargreaves test of mice after CCI surgery. n.s. not significant. Two-way ANOVA test followed by Sidak’s post hoc test. n=6 mice/group. (F) The schematic diagram of scRNA or si*Vdac3* DRG injection, and the workflow of CCI surgery and behavioral test. (G-J) Paw withdrawal frequency to 0.4 g, 0.6 g, and 1.0 g von Frey filaments and withdrawal latency in Hargreaves test of mice after CCI surgery. n.s. not significant. Two-way ANOVA test followed by Sidak’s post hoc test. n=6 mice/group.

**Figure S9.** **Genetic strategies for downregulating VDAC1 expression.** (A) Western blot analysis of VDAC1 expression in isolated primary sensory neurons from mice receiving scRNA or *siVdac1*. **p<0.05*. Student’s t test. n=3 mice/group. (B) Western blot analysis of VDAC1 expression in L3-L5 DRGs from Sham and CCI *Vdac1 (+/-)* mice. n.s. not significant. Student’s t test. n=3 mice/group.

**Figure S10.** **MMP of DRG neurons is increased after capsaicin stimulation, and it can be reversed by *Vdac1* knock-down.** (A) Representative JC-1 staining images of neurons from naïve *wt* mice after Vehicle or Cap incubation (2 μM, 30 min). Scale bar: 10 μm. (B) Quantitative analysis of neuronal MMP accessed by the intensity ratio of JC-1 aggregates (red) to monomer (green) of neurons from naïve *wt* mice after Vehicle or Cap incubation. **p<0.05*. Student’s t test. n=3 mice/group. (C) Representative JC-1 staining images of neurons from scRNA or *siVdac1* group after Cap incubation. Scale bar: 10 μm. (D) Quantitative analysis of neuronal MMP accessed by the intensity ratio of JC-1 aggregates (red) to monomer (green) of neurons from scRNA or *siVdac1* group. **p<0.05*. Student’s t test. n=3 mice/group.
